# Risks of Zoonotic Transmission of COVID-19 During Eid-Ul-Adha in Pakistan

**DOI:** 10.1017/dmp.2020.278

**Published:** 2020-07-27

**Authors:** Tauqeer Hussain Mallhi, Yusra Habib Khan, Muhammad Hammad Butt, Aroosa Liaqat, Arooj Abid, Abrar Ahmad, Shahzadi Misbah

**Affiliations:** Department of Clinical Pharmacy, College of Pharmacy, Jouf University, Sakaka, Al-Jouf, Kingdom of Saudi Arabia; Faculty of Pharmacy, University of Central Punjab, Lahore, Pakistan; University College of Pharmacy, University of the Punjab, Lahore, Pakistan; Tehsil Headquarter Hospital, Kotli Sattian, Rawalpindi, Pakistan

**Keywords:** COVID-19, SARS-CoV-2, Eid-Ul-Adha, recautionary measures, zoonotic transmission

Pakistan is expected to celebrate Eid-ul-Adha, an annual religious festival during which millions of farm animals, including sheep, goats, cows, buffalo, and camels are sacrificed, in the end of July or early August this year. The sacrifice of animals is controlled and confined to designated places in many Muslim countries. However, the celebration of festive and slaughter practices markedly differ in Pakistan amid various cultural and religious beliefs.^1^ These practices include the advance purchase of animals, keeping animals at homes, roaming around with purchased animals, lack of health checks on animals, congested and crowded livestock markets, mass migration of animal traders, increased public movements for animal buying, non-professional butchers, in-house slaughtering, self-sacrifice of animals, gathering of spectators around the butcher to watch the slaughter, mass in-house gatherings for at least 1 week, and increased home visits for Eid greetings.^2^ Since a recent investigation found the potential of zoonotic transmission of coronavirus disease (COVID-19) by farm animals,^3^ we felt inclined to underscore the risks of virus transmission from humans to animals due to various activities surrounded by the festive celebration in Pakistan.

There is a probability that severe acute respiratory syndrome coronavirus 2 (SARS-CoV-2) may have evolved to infect many species of farm animals. It must be noted that the SARS epidemic in 2003 was also originated from bats, but it transmitted to human via a variety of intermediate hosts, including masked palm civet cats and raccoon dogs. Moreover, the outbreak of the highly fatal Middle East respiratory syndrome coronavirus (MERS-CoV) was also linked to contact with dromedary camels.^4^ A team of researchers at the University of Hunan have studied the lung structures of 251 different animals to ascertain their capabilities to contract SARS-CoV-2.^3^ Their findings suggest that the virus has evolved the ability to infect many animals, including cats, cows, goats, pigs, sheep, buffalo, and pigeons. These results raised serious concerns of virus transmission from human to these mammals, which can act as intermediate hosts for disease spread. Since native reservoir hosts of viruses, such as bats, usually live far away from human community, intermediate hosts can play critical roles in transmitting viruses to humans, resulting in future epidemics. A phylogenetic analysis and critical site comparison of angiotensin converting enzyme 2 (ACE2) has been used to predict the intermediate hosts of SARS-CoV-2. Alarmingly, ACE2s of the pangolin, cat, cow, buffalo, goat, and sheep were ranked as the top mammals that could be potentially infected by SARS-CoV-2, while murine ACE2s scored the lowest. It is pertinent to mention that ACE2s in animals, which are abundantly sacrificed during the Eid-Ul-Adha, have the ability to contract SARS-CoV-2, like humans.^3^ Considering the widespread existence of these animals, some of them might serve as intermediate hosts for SARS-CoV-2, which called for the attention in disease control. Although screening of intermediate hosts will be the logical measure, such mass screening seems impractical and expensive during such a short time before the festive celebrations, particularly in resource limited countries like Pakistan. The only possible approach would be cautionary measures during the sale, purchase, and sacrifice of animals.

The lessons learned from past episodes of MERS-CoV and SARS-CoV are being exploited to report this new virus (SARS-CoV-2). We believe that risks of zoonotic transmission of COVID-19 should be considered during the preparation of festive celebrations, and immediate measures must be taken to avoid any possible surge in COVID-19 cases during the Eid-Ul-Adha.

Since the general public, staff at abattoirs, and animal handlers might not be aware of virus tendency to infect farm animals, effective educational campaigns through electronic media will be of utmost importance. Animal purchases must be subjected to strict controls, and online purchases must be encouraged to the extent possible to reduce crowds in the livestock markets. In addition, the regular use of a disinfectant solution as a spray or cloth wipe will be helpful to avoid the fomite transmission.

Health authorities should consider the prohibitions on in-house slaughtering, and sacrifice should be limited to the abattoirs. The movements of people with their animals and social gatherings must be controlled by the district level administration. Public health officials should encourage the staff at abattoirs to follow the good slaughtering practices through structured awareness and training campaigns. Since there are several precautionary notes on the transmission of MERS-CoV through uncooked meat, thorough washing of meat before use and storage must be practiced. Moreover, a recent viewpoint urges that the possibility of SARS-CoV-2 also as a food-borne infection that is transmitted by the respiratory route cannot be rejected entirely.^5^ Health authorities must advise the general population to always thoroughly wash and cook meat. As mass screening of animals and population is beyond the testing capacity of the country; post-festival surveillance programs need to be in place for a timely identification of variations in pattern of incident cases.
